# Deep Learning for Pathology: YOLOv8 with EigenCAM for Reliable Colorectal Cancer Diagnostics

**DOI:** 10.3390/bioengineering12111203

**Published:** 2025-11-03

**Authors:** Mohamed Farsi, Hanaa ZainEldin, Hanaa A. Sayed, Rasha F. El-Agamy, El-Sayed Atlam, Shatha Abed Alsaedi, Majed Alwateer, Hossam Magdy Balaha, Mahmoud Badawy, Mostafa A. Elhosseini

**Affiliations:** 1 Department of Information Systems, College of Computer Science and Engineering, Taibah University, Yanbu 46421, Saudi Arabia; mafarsi@taibahu.edu.sa; 2 Computers and Control Systems Engineering Department, Faculty of Engineering, Mansoura University, Mansoura 46421, Egypt; eng_hanaa@mans.edu.eg (H.Z.); hmbala01@louisville.edu (H.M.B.); engbadawy@mans.edu.eg (M.B.); 3 Department of Computer Science, College of Computer Science and Engineering, Taibah University, Yanbu 46421, Saudi Arabia; hasali@taibahu.edu.sa (H.A.S.); relagamy@taibahu.edu.sa (R.F.E.-A.); satlam@taibahu.edu.sa (E.-S.A.); saasaedi@taibahu.edu.sa (S.A.A.); mwateer@taibahu.edu.sa (M.A.); 4 Department of Computer Science, Faculty of Computers and Information, Assiut University, Assiut 71516, Egypt; 5 Computer Science Department, Faculty of Science, Tanta University, Tanta 31527, Egypt; 6 Department of Computer Science, Tanta University, Tanta 31527, Egypt; 7 Bioengineering Department, J.B. Speed School of Engineering, University of Louisville, Louisville, KY 40292, USA; 8 Department of Computer Science and Information, Applied College, Taibah University, Medinah 41461, Saudi Arabia

**Keywords:** cancer diagnosis, deep learning (DL), explainable artificial intelligence (XAI)

## Abstract

Colorectal cancer (CRC) is one of the most common causes of cancer-related deaths globally, making a timely and reliable diagnosis essential. Manual histopathology assessment, though clinically standard, is prone to observer variability, while existing computational approaches often trade accuracy for interpretability, limiting their clinical utility. This paper introduces a deep learning framework that couples the YOLOv8 architecture for multiclass lesion classification with EigenCAM for transparent model explanations. The pipeline integrates three core stages: (i) acquisition and preprocessing of 5000 hematoxylin-and-eosin-stained slides from the University Medical Center Mannheim, categorized into eight tissue types; (ii) comparative evaluation of five YOLOv8 variants (Nano, Small, Medium, Large, XLarge); and (iii) interpretability through EigenCAM visualizations to highlight discriminative regions driving predictions. Extensive statistical validation (including box plots, empirical cumulative distribution functions, Bland–Altman plots, and pair plots) demonstrated the robustness and reliability of the framework. The YOLOv8 XLarge model achieved 99.38% training accuracy and 96.62% testing accuracy, outperforming recent CNN- and Transformer-based systems (≤95%). This framework establishes a clinically dependable foundation for AI-assisted CRC diagnosis by uniting high precision with visual interpretability. It represents a significant step toward real-world deployment in pathology workflows.

## 1. Introduction

Colorectal cancer (CRC) ranks as one of the most prevalent and dangerous cancers worldwide, constituting a significant burden on public health systems. It originates from the unregulated proliferation of atypical cells in the colon or rectum, usually commencing as benign polyps that may evolve into cancer over time. The incidence of CRC exhibits considerable geographical and demographic disparities, with higher rates observed in developed countries than in developing regions. Lifestyle variables, including nutrition, physical inactivity, obesity, smoking, and excessive alcohol use, substantially elevate the risk of CRC, underscoring the need for preventive strategies in mitigating its prevalence [1].

Traditionally, CRC is diagnosed using screening methods such as colonoscopy, sigmoidoscopy, and imaging tests like computed tomography (CT) colonography. Colonoscopy represents the highest standard for CRC detection, enabling direct vision of the colon’s interior and allowing for the excision of polyps for biopsy. However, the complexity of tissue structures and the potential for human error in identifying and classifying abnormal cells through histopathological analysis present ongoing challenges. These limitations often lead to diagnostic delays and variability in accuracy, underscoring the need for more reliable and faster diagnostic approaches, mainly as CRC in younger populations is frequently diagnosed at more advanced stages [2]. Early detection through screening is crucial for enhancing CRC outcomes as it facilitates the identification and removal of precancerous polyps or early-stage tumors. Screening modalities encompass a range of tests, including colonoscopy, stool DNA testing, and flexible sigmoidoscopy, each with advantages and limitations. However, disparities in access to screening services and low adherence rates continue to hinder efforts to achieve widespread screening coverage [3].

Given the critical importance of early detection in improving CRC prognosis, there is an increasing interest in utilizing artificial intelligence (AI) and deep learning (DL) technologies to improve diagnostic accuracy and effectiveness. Recent developments in AI demonstrate significant potential to automate the detection and categorization of CRC by analyzing medical photographs, particularly histopathology slides. However, while these technologies offer substantial benefits, they present challenges, particularly in model interpretability and clinical trust. YOLOv8 (You Only Look Once, version 8) is a cutting-edge object detection model for detecting and categorizing CRC in medical imaging. Unlike standard convolutional neural networks (CNNs), which focus on image classification, YOLOv8 is engineered for instantaneous object recognition. It is ideal for detecting specific malignant spots in high-resolution histopathology images. This capability enables more precise localization and classification of tumor tissues, thereby increasing CRC diagnosis accuracy and speed. YOLOv8 was chosen for CRC classification because of its capacity to effectively analyze high-resolution histopathological images, detect multiple regions of interest, and accurately identify malignant tissues. YOLOv8 is distinguished from other DL models by its real-time ability to recognize objects. Although powerful for some image classification tasks, Vision Transformers usually require more significant computational resources and longer processing times, making them less viable for fast, real-time applications like CRC detection [4].

YOLOv8 has significant advantages in CRC classification, including detecting and categorizing small, irregular tumor areas with high accuracy. It also has faster training and inference times than other models, keeping it appropriate for real-time operational applications. Moreover, its architecture is designed to efficiently handle large, high-resolution histopathology images, essential for precise medical diagnosis. However, these benefits are not without costs. YOLOv8 requires significant computational resources, particularly during the training phase, which may limit its usability in environments with less advanced infrastructure. Additionally, the model’s efficacy is predominantly contingent upon the accessibility of extensive, annotated datasets, which are notoriously difficult to obtain in medical applications. Despite these challenges, YOLOv8’s ability to balance speed, accuracy, and efficiency makes it an excellent option for CRC classification, as it overcomes many of the constraints of manual and slow diagnostic methods by providing exact, real-time detection of malignant regions.

As AI models become more crucial to medical diagnosis, the demand for interpretability has increased dramatically. Recognizing the decision-making processes of AI models is essential in high-stakes domains such as CRC detection, enabling clinicians to trust and act on the model’s output. Interpretability tools, such as EigenCAM, offer visual insights into decision-making, allowing healthcare practitioners to test and evaluate model predictions more easily. EigenCAM was chosen for this investigation due to its simplicity and effectiveness. It generates heat maps that graphically highlight areas of the image that are most critical to a model’s classification result, providing intuitive feedback on the model’s attention during diagnosis. These heatmaps are particularly effective in histological images for identifying tumor areas, which are crucial in CRC diagnosis. This transparency allows physicians to understand which areas the model considers significant, thereby increasing trust in the AI’s conclusions [5].

Given the significant challenges associated with traditional CRC diagnostic methods, including the complexity of histopathological analysis and the potential for human error, there is an imperative demand for more dependable, precise, and effective diagnostic instruments. The increasing complexity and volume of medical imaging data further underscore the necessity for automated systems that help to improve diagnostic accuracy while alleviating the workload of healthcare practitioners. In this context, integrating YOLOv8 with interpretability tools like EigenCAM is not just an enhancement but a necessity. This method enhances the accuracy and efficiency of CRC diagnosis. It ensures that these advancements are accessible and trustworthy to the clinicians who rely on them. As we move towards more sophisticated AI-driven diagnostic systems, the combination of robust performance with clear interpretability will be key to transforming CRC diagnosis and ultimately improving patient care. This study aims to create and assess a sophisticated AI-driven framework for classifying and interpreting CRC histopathology pictures. This study presents several key contributions:

- YOLOv8 for CRC Classification: Apply the YOLOv8 model to CRC histopathology images, achieving superior classification accuracy, critical for improving diagnostic precision and patient outcomes.
- EigenCAM for Interpretability: Enhanced model transparency by integrating EigenCAM, providing visual explanations of predictions, and increasing the model’s interpretability and clinical trust.
- Comprehensive Pipeline: Design and implement a complete workflow, covering all stages from data acquisition to evaluation, using a dataset of 5000 CRC histopathology slides. This ensures that the proposed framework is robust and applicable in practical settings.
- Improved Performance: Extensive statistical analysis was conducted using box plots, empirical cumulative distribution functions (ECDFs), Bland–Altman plots, and pair plots. Achieving a testing accuracy of 96.62%, demonstrating the effectiveness of combining YOLOv8 with interpretability tools in CRC diagnostics.

The rest of this paper is structured as follows: the related studies are reviewed in Section 2 examines particular studies and methods in classifying and interpreting CRC histopathology images. Section 3 explores the suggested study technique, incorporating the utilization of YOLOv8 and EigenCAM for CRC diagnosis. Section 4 presents the results of this study, demonstrating how the proposed methodologies address the stated deficiencies and enhance the progression of CRC diagnosis. Section 5 summarizes the study.

## 2. Related Works

Many studies utilize YOLO versions, Transformers, and CNNs for CRC diagnosis, classification, and the application of interpretability tools. Lalinia et al. [6] introduced a polyp detection technique utilizing AI and the YOLOv8 network, attaining equilibrium between accuracy and computational effectiveness. Moreover, Palanivel et al. [7] used DL to assess the efficacy of the YOLOv8 for diagnosing multiple cancer types. YOLOv8, distinguished for its real-time object identification abilities, is an excellent choice for automating classifying malignant areas in medical photographs. Several YOLO-based target detection approaches have been introduced to enhance polyp detection accuracy. Guo et al. developed an automated polyp detection system utilizing the YOLOv3 architecture integrated with active learning to reduce false positive rates in polyp identification [8]. The authors in [9] integrated a feature extraction and fusion mechanism into the YOLOv3 network to obtain feature maps at both high and low levels. A real-time identification approach utilizing YOLOv4 was proposed in [10]. It integrates the CSPNet architecture, Mish activation function, Distance-Intersection over Union (DIoU) loss function, and a Transformer block to enhance accuracy and performance. Improvements in YOLO-based target detection algorithms are expected to improve the efficiency of polyp detection in colonoscopy operations.

Lee and his team [11] built a real-time polyp identification system employing YOLOv4, incorporating a multiscale mesh to detect diminutive polyps. The system’s efficiency improved by integrating advanced data augmentation methods and various activation functions. The authors in [12] presented a YOLOv5-based model for real-time polyp classification that included a self-attention mechanism, enhancing important features while reducing irrelevant ones to boost detection accuracy. Pacal et al. [13] employed the Scaled YOLOv4 method to assess new datasets, SUN and PICCOLO. Durak et al. [14] trained innovative object detection algorithms [15,16,17], for the automated diagnosis of gastric polyps.

Researchers have also employed Vision Transformers for multiclass tissue classification on CRC histology datasets [18]. Some studies have introduced methods such as MoViT to tackle data limitations, achieving near full-dataset performance using just 1–3% of the data [19]. This approach not only outperforms alternative techniques but also markedly decreases training duration. However, concerns arise regarding the model’s generalizability and the potential for overfitting when relying on such a small dataset portion, potentially limiting its effectiveness on new, unseen data. Another strategy to enhance predictive accuracy is integrating multimodal data. By combining histopathological images with genomic data, Transformer-based models like TransSurv have improved predictions of patient survival rates, achieving a concordance index (C-index) of 0.822 using the NCT biobank dataset [20]. This integrated approach highlights the potential of using multiple data sources to strengthen diagnostic and prognostic models in CRC.

Hybrid models fusing CNNs and Transformers’ strengths have also been explored. These models have improved over other approaches by utilizing CNNs for local feature extraction and Transformers for acquiring global contextual information from photographic patches [21]. Innovations such as removing the class token layer have contributed to performance gains. However, these modifications introduce additional complexity, affecting model training efficiency and interpretability.

Advancements in CRC histopathology image classification have been achieved through enhanced methods utilizing architectures like ResNet-50 with transfer learning and fine-tuning techniques [22]. These models have significantly improved classification accuracy, representing significant progress over earlier studies. Nonetheless, challenges remain in generalizing these models to external datasets, where performance may decline. Further innovations include models like TransNetV, which utilize CNNs’ local feature extraction capabilities and pass these features through the Transformer’s attention mechanisms to capture global context [23]. This methodology capitalizes on CNNs’ weight-sharing properties and Transformers’ ability to understand spatial relationships. It is appropriate for varied datasets and complicated feature analysis.

To enhance detection accuracy, CRC detection networks incorporating coordinate attention transformers and atrous convolution have been proposed [24]. These networks first denoise input histopathology images using filters to preserve essential features. Novel modules combining local and global information enable the classification of colorectal tissue at different scales. Attention models such as the Cross-shaped Window (CrSWin) Transformer also capture subtle changes in colorectal tissue from multiple perspectives. Comparative studies have highlighted the performance disparities between different models. For instance, evaluations comparing Vision Transformer models (ViT-B/16, ViT-B/32, ViT-L/16) with YOLOv8 variants (YOLOv8n-cls, YOLOv8s-cls, YOLOv8m-cls) have shown that YOLOv8 outperforms ViT in both training and testing sets [25]. Several factors may contribute to this disparity. Overfitting is a potential issue with ViT models; their complexity may cause them to learn noise and irrelevant features from the training data, resulting in inadequate generalization to novel data. Moreover, ViT models may require more precise hyperparameter tuning due to their intricate architectures, whereas YOLOv8 models benefit from optimized pretrained hyperparameters that are robust across various training conditions.

The inherent complexity of Transformer-based models, designed initially for Natural Language Processing tasks, can present challenges in computer vision applications like CRC diagnosis. Their sophisticated architecture often necessitates large training datasets to achieve optimal accuracy. In contrast, YOLOv8 models, with their lower complexity, can achieve high accuracy even with modestly sized training datasets [25]. This makes them more suitable for medical imaging scenarios where data availability is limited. CNNs have been extensively employed in categorizing and grading CRC tissues using histology datasets such as the Kather CRC dataset [26]. This dataset has facilitated numerous studies aiming to evaluate the effectiveness of different models and determine feasible strategies for enhancing the precision and dependability of CRC detection. Early explorations involved training and testing multiple CNN architectures, including AlexNet [27], VGGNet [28], and GoogLeNet [29], to evaluate their performance in CRC classification [30]. These efforts illustrated the capabilities of CNNs in medical image analysis, facilitating the development of more advanced models.

Expanding on these foundations, researchers have explored the versatility of CNNs across different types of cancer. For instance, models trained on histology datasets have been applied to identify invasive ductal carcinoma in breast cancer images, showcasing CNNs’ adaptability in histopathological analysis [31]. Attention-based CNN models have been developed to enhance classification performance for grading CRC histology images, integrating mechanisms that allow models to focus on relevant regions within the images [31]. Comparative studies have assessed multiple machine-learning approaches on the Kather colon histological dataset, including K-Nearest Neighbor [32], Random Forest, Logistic Regression, and CNNs [33]. These investigations consistently found CNNs to be the most effective strategy, highlighting their superior capability in feature extraction and classification tasks. Further advancements were achieved by combining CNN architectures with transfer learning techniques to identify multiple tissue types observed in CRC examinations automatically [34]. Researchers enhanced classification performance by adapting CNN structures to extract features and integrating them with machine-learning algorithms.

To address geometric variability in histological pictures, efforts have focused on retrieving local elements such as architectural, geometric, and energetic information and patterns generated from the Riesz transform and monogenic local binary patterns [35]. Utilizing these features on multiclass histology datasets like Kather [26] and Kimiapath [36] has improved the models’ generalization ability across diverse data sources. Transfer learning has emerged as a critical strategy to improve CNN models, especially when data scarcity poses challenges. DL models incorporating transfer learning and attention mechanisms have been developed to estimate complex patterns, such as electromyography hand movements, demonstrating the versatility of these approaches [35]. The optimization framework in transfer learning emphasizes what, how, and when to transfer and “from where to transfer”, highlighting the importance of source selection in effective knowledge transfer [37].

In CRC tissue classification, methods utilizing multispectral histopathological imaging (HI) have identified tissue types associated with CRC varieties, including benign hyperplasia (BH), intraepithelial neoplasia (IN), and carcinoma [38]. Combining HI analysis with feature assessment has led to reliable computer-aided diagnosis (CAD) techniques for metastatic lymph nodes (LNM) in CRC, enhancing diagnostic accuracy [39]. DL models based on CNN structures have been pivotal in distinguishing CRC tissue from various datasets, underscoring CNN’s critical role in medical image analysis.

Introducing interpretability tools shows a stronger dedication to model transparency and validation in clinical environments. Several Class Activation Mapping (CAM) methods have been introduced, such as CAM [40], GradCAM [41], GradCAM++ [42], XGradCAM [43], AblationCAM, EigenGrad-CAM, Layer-CAM [44], and FullGrad [45], to provide high-resolution, class-discriminative explanations of CNN outcomes. While GradCAM remains the leading technique in the medical field [46], the development of alternatives like EigenCAM shows ongoing attempts to enhance interpretability.

Activation mapping techniques accomplish several objectives, such as providing frameworks for developing new mapping strategies, assisting in model verification and comparison, improving the interpretability of novel models, acting as explanatory tools for CNNs, and creatively choosing features. Activation mapping techniques accomplish several objectives, such as providing frameworks for developing new mapping strategies, helping with model validation and evaluation, improving the interpretability of novel models, acting as explanatory tools for CNNs, and creatively choosing features. By weaving these approaches into a cohesive strategy, the medical imaging community continues to advance the reliability and effectiveness of AI models in CRC classification and beyond.

Despite the importance of interpretability in medical AI, the lack of tools like EigenCAM in the co-occurrence network highlights a significant gap in current research. EigenCAM is a useful interpretability method that visually explains model predictions by highlighting regions in medical images most essential to the model’s decision-making process. This is especially important in CRC diagnosis, where knowing the “why” of a model’s classification can boost clinician confidence and decision-making. The lack of research focusing on EigenCAM suggests untapped potential for combining this interpretability tool with advanced models such as YOLOv8 to ensure that AI systems are accurate, transparent, and therapeutically relevant.

This study aims to enhance the interpretability and practical application of YOLOv8 in the detection, diagnosis, and classification of CRC, thereby contributing to the evolving field of medical AI. This technique will connect advanced AI models with clinical applications, ensuring that AI-driven diagnostic tools are effective and reliable for healthcare professionals. Our proposed research addresses critical gaps by employing YOLOv8 for CRC classification and improving model transparency through EigenCAM.

## 3. Methodology

**Figure 1:**
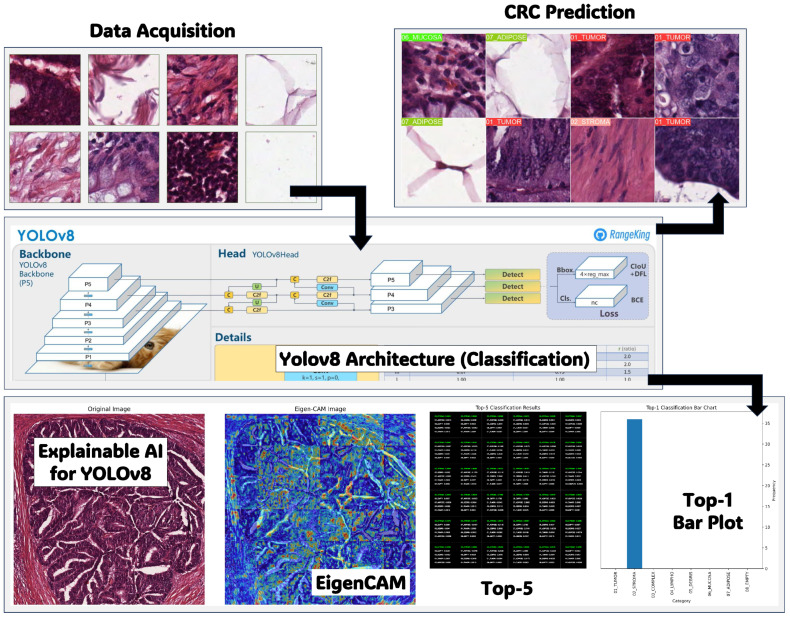
Proposed framework for CRC diagnosis and interpretation (integrating YOLOv8 for automated lesion classification and EigenCAM for enhanced model interpretability) where it utilizes H&E-stained histopathology slides, ensuring a robust and clinically relevant approach to CRC diagnostics. That end-to-end pipeline exhibits how advanced AI models can combine with interpretability tools to achieve seamless integration and thus build faith in users and usability by clinicians.

As depicted in Figure 1, the proposed framework offers a comprehensive approach to CRC diagnosis and interpretation using histopathology data. This framework integrates multiple methodologies, including data acquisition and preprocessing, YOLOv8 classification for CRC prediction, EigenCAM interpretability, and performance evaluation. The study used a database comprising hematoxylin-and-eosin-(H&E) stained histopathology slides of CRC samples for data acquisition and preprocessing. These slides were systematically captured to form a dataset of images categorized into various stages of CRC. The YOLOv8 classification method was employed for CRC prediction, aiming to classify histopathology slides into different stages of CRC accurately. YOLOv8, known for its object detection capabilities, incorporates architectural enhancements such as anchor-free detection, new convolutions, and mosaic augmentation. Anchor-free detection eliminates predefined anchor boxes, while new convolutions optimize feature extraction. Mosaic augmentation improves model robustness by presenting it with varied contexts. The study utilized the Ultralytics YOLOv8 package, integrating pretrained classification models.

Interpretability using EigenCAM offers insights into classification tasks by generating Class Activation Maps (CAM) to discern influential pixels or regions in images. EigenCAM facilitates efficient interpretation without requiring modifications, seamlessly integrating with CNN models. It employs Singular Value Decomposition (SVD) to derive Class Activation Maps, enabling visualization of significant image regions that contribute to classification decisions. Performance evaluation of CRC classification employed various metrics that provide complete comprehension of classification models’ effectiveness, considering aspects such as model accuracy, sensitivity, specificity, and overall performance on imbalanced datasets.

### 3.1. Data Acquisition and Preprocessing

The framework acquired ten anonymized H&E-stained CRC tissue slides from the pathology repository at https://zenodo.org/records/53169 (accessed on 10 January 2025). The research examined eight categories of tissue: tumor epithelium, simple stroma, complex stroma, immune cells, debris, normal mucosal glands, adipose tissue, and background. The 5000 generated images constituted the training and testing dataset for the classification issue.

**Figure 2:**
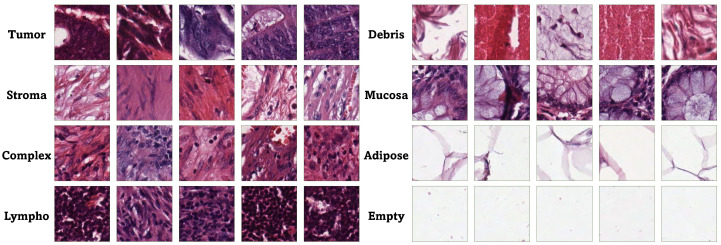
Dataset samples of the H&E-stained histopathology slides. The images demonstrate the diversity and complexity of CRC lesions, serving as a basis for training and evaluating the YOLOv8 model. The high-quality annotations in the dataset guarantee the reliability and generalizability of the proposed diagnostic framework.

Patient selection and characteristics were anonymized, adhering to ethical considerations. Imaging techniques involved manual annotation and tessellation of tissue areas to extract representative images, ensuring a comprehensive representation of CRC histopathology. The study’s ethical considerations prioritized patient confidentiality and consent, with data availability restricted to anonymized datasets for research purposes [47]. Figure 2 displays instances from the dataset utilized in this paper.

The 5000 generated images constituted the training and testing dataset for the classification task. To ensure robust evaluation, the dataset was partitioned using an 80:20 stratified split, yielding 4000 training images and 1000 test images, with proportional representation across all eight tissue categories.

### 3.2. Classification and Parameters Tuning Using YOLOv8

The detection and diagnosis of CRC typically require the classification of lesions into different categories, such as stroma, lympho, adipose, and tumor. YOLOv8 can function as a classification model by modifying its output layer to estimate probabilities for every class label. Using a dataset marked with CRC lesion categories, YOLOv8 can effectively learn to classify lesions into the defined groups, allowing clinicians to recognize and prioritize areas of interest for additional assessment and reporting [48].

YOLOv8 includes a deep neural network structure that employs convolutional layers to retrieve features from input images. Subsequently, these features are analyzed through multiple layers to forecast bounding boxes and related class probabilities. YOLOv8 generally uses a modified backbone network, commonly derived from DarkNet or ResNet architectures, to improve feature extraction and representation [49].

The principal feature of YOLOv8 is its prediction methodology, which utilizes a grid layout. The input image is divided into a grid, with each cell responsible for predicting bounding boxes and class probabilities for the items included. This grid-based approach enables YOLOv8 to efficiently handle objects of varying sizes and aspect ratios inside a single image. Although YOLOv8 immediately provides remarkable performance, adjusting its parameters can improve its efficiency for particular uses like our research [50].

YOLOv8 utilizes a CNN as its foundational architecture for extracting features. The CNN comprises various layers, including convolutional, pooling, and activation functions that convert the input picture into a feature map. The bounding box prediction in YOLOv8 includes determining the coordinates (center (x,y), width *w*, and height *h*), confidence scores, and class probabilities for every bounding box. These predictions are improved iteratively during training by employing techniques like gradient descent.

Following the prediction of bounding boxes, YOLOv8 implements non-max suppression to eliminate redundant bounding boxes and keep only the most confident ones. This aids in removing duplicate detections of the identical object. YOLOv8 employs a combination of localization loss, confidence loss, and classification loss to train the network. Equation (1) illustrates the loss function for YOLOv8.

(1) $$\begin{array}{cc} L= & \lambda_{coord}\times \sum_{i=0}^{S^{2}}\sum_{j=0}^{B}1_{ij}^{obj}\left[{\left(x_{i}-{\hat{x}}_{i}\right)}^{2}+{\left(y_{i}-{\hat{y}}_{i}\right)}^{2}\right]+\\ & \lambda_{coord}\times \sum_{i=0}^{S^{2}}\sum_{j=0}^{B}1_{ij}^{obj}\left[{\left(w_{i}-{\hat{w}}_{i}\right)}^{2}+{\left(h_{i}-{\hat{h}}_{i}\right)}^{2}\right]+\\ & \lambda_{noobj}\times \sum_{i=0}^{S^{2}}\sum_{j=0}^{B}1_{ij}^{noobj}{\left(C_{i}-{\hat{C}}_{i}\right)}^{2}+\\ & \sum_{i=0}^{S^{2}}1_{i}^{obj}\sum_{c\in \mathrm{classes}}{\left(p_{i}\left(c\right)-{\hat{p}}_{i}\left(c\right)\right)}^{2} \end{array}$$

where:

- $\lambda_{coord}$ and $\lambda_{noobj}$: Hyperparameters controlling the importance of localization and confidence losses, respectively.
- *S*: The number of grid cells within the image.
- *B*: The quantity of bounding boxes anticipated for each grid cell
- $1_{ij}^{obj}$: one if the *j*-th bounding box in cell *i* contains the center of a ground-truth object and zero otherwise.
- $1_{ij}^{noobj}$: One if the *j*-th bounding box in cell *i* does not contain the center of a ground-truth object, and zero otherwise.
- $\left(x_{i},y_{i}\right)$: Predicted coordinates of the center of the bounding box.
- $\left(w_{i},h_{i}\right)$: Predicted width and height of the box’s perimeter.
- $C_{i}$: Predicted confidence score for the box’s perimeter.
- $p_{i}\left(c\right)$: Predicted probability of class *c* for the bounding box.

### 3.3. Performance Evaluation

The classification models were comprehensively assessed with various metrics to determine their efficiency in classifying CRC tissue. These metrics encompassed critical elements of classification performance, such as Accuracy, Precision, Recall, Specificity, F1 score, Intersection over Union (IoU), Balanced Accuracy (BAC), Matthews correlation coefficient (MCC), Youden’s Index, Yule’s Q, and the average performance across all metrics [51,52].

Accuracy ($\frac{TP+TN}{TP+TN+FP+FN}$) reflected the ratio of accurately classified instances compared to the total. At the same time, Precision ($\frac{TP}{TP+FP}$) and Recall ($\frac{TP}{TP+FN}$) offered details about the model’s capacity to provide accurate predictions and identify all pertinent positive instances, respectively. Specificity ($\frac{TN}{TN+FP}$) evaluated the model’s capacity to recognize negative instances [53] accurately.

The F1 score ($2\times \frac{\mathrm{Precision}\times \mathrm{Recall}}{\mathrm{Precision}+\mathrm{Recall}}$), the harmonic mean of Precision and Recall, provided a balanced overall performance assessment. IoU assessed the overlap between predicted and actual areas, providing a thorough perspective on spatial classification accuracy. It can be calculated using $\frac{\left|P\cap G\right|}{\left|P\cup G\right|}$ where *P* is the set of predicted positive pixels and *G* is the set of ground-truth positive pixels. BAC ($\frac{\mathrm{Recall}+\mathrm{Specificity}}{2}$) reflected the mean of sensitivity and specificity, offering a more detailed evaluation of the model’s effectiveness on positive and negative classes [54]. MCC is the correlation coefficient between observed and predicted binary classifications, ranging from −1 to +1 and can be calculated using $\mathrm{MCC}=\frac{TP\cdot TN-FP\cdot FN}{\sqrt{\left(TP+FP)(TP+FN)(TN+FP)(TN+FN\right)}}$.

Youden’s Index (Recall+Specificity−1) gauges the model’s ability to avoid false positives and negatives simultaneously. At the same time, Yule’s Q ($\frac{TP\times TN-FP\cdot FN}{TP\times TN+FP\cdot FN}$) quantifies the strength and direction of association between predicted and actual classifications. Finally, Mean Performance across all metrics provided an overarching evaluation, consolidating the model’s performance into a single metric for a comprehensive assessment.

### 3.4. YOLOv8 Explainability Using EigenCAM

Deep learning models are inherently opaque and lack human-interpretable logic. Therefore, employing model explainability tools becomes imperative to extract meaningful insights from these complex systems [55]. In computer vision, where models can be immensely intricate with millions of parameters and numerous layers, deciphering pertinent information for interpretation presents a formidable challenge. A pivotal objective of model explainability in computer vision is discerning the visual features or spatial regions pivotal to the model’s predictions. This becomes particularly significant in tasks such as CRC classification, where prediction outputs often lack granularity, hindering the identification of essential features.

EigenCAM emerges as a potent model explainability tool tailored for CNNs, a cornerstone architecture in computer vision. Introduced in 2020 by Muhammad et al. [40], EigenCAM builds upon Class Activation Maps (CAM), facilitating an understanding of the visual features learned by the model to make predictions. These Class Activation Maps offer intuitive visualizations aligning with human perception, enabling users to correlate model insights with original image contents effectively [5].

Unlike other techniques like GradCAM [56], EigenCAM sets itself apart with its straightforward implementation and smooth integration without the need for retraining or changes to layers. EigenCAM produces heatmaps that emphasize areas of peak activation by calculating and visualizing the main components of the extracted features from convolutional layers, providing insights into where the model is concentrating its attention across various layers.

### 3.5. Overall Framework Algorithm

As discussed, the proposed framework integrates advanced DL techniques with interpretability tools to address challenges in CRC diagnosis. Algorithm 1 outlines the end-to-end process ensuring a robust and clinically relevant approach. By utilizing YOLOv8 for automated lesion classification and EigenCAM for enhanced interpretability, the framework aims to provide clinicians with reliable, transparent, and user-friendly CRC detection and classification tools. It details the step-by-step methodology, highlighting key components such as data acquisition, model initialization, training, inference, and performance evaluation.  
**Algorithm 1:** Pseudocode for CRC Diagnosis Framework Using YOLOv8 and EigenCAM
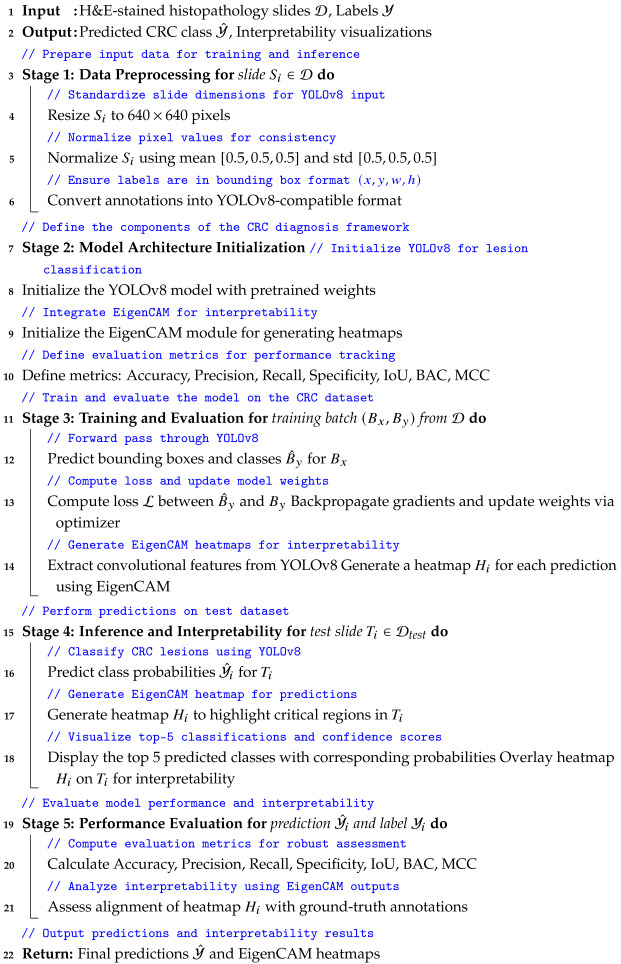


## 4. Experiments and Discussion

The experiments were conducted on a Windows 11 (64-bit) system equipped with an Intel Core i7-1165G7 processor (four cores, eight threads, 2.8 GHz base, 4.7 GHz boost), eight gigabytes of DDR4 RAM, and a 512-gigabyte NVMe SSD for storage. The software environment was designed around Python 3.10, with Jupyter Notebook v7 as a development tool. NumPy and Pandas were important libraries for data processing, Scikit-Learn, TensorFlow 2.9, and PyTorch 1.12 for machine learning, and Matplotlib 3.10.7 and Seaborn 0.13.2 for data visualization. Nine trials were applied to the different experiments to report the statistical analysis. All experiments were conducted on a dataset of 5000 CRC histopathology tiles, split 80% for training (4000 images) and 20% for testing (1000 images) via stratified sampling to maintain class balance. Nine independent trials were performed to report statistical variability.

Table 1 illustrates the performance metrics of YOLOv8 across various architectures and dataset subsets, emphasizing training and testing phases. During the training stage, all models demonstrate remarkably high performance, with accuracies between 99.76% and 99.81%. Precision, recall, specificity, F1 score, IoU, BAC, MCC, Youden’s index, Yule’s Q, and mean scores consistently show strong performance across all architectures, indicating a high model capability in detecting and classifying objects within the training dataset.

As we move into the testing phase, the models show high accuracy, though slightly reduced compared to the training phase, which is anticipated because of the unfamiliarity of the test data. Nonetheless, the accuracy stays over 98.81% for all architectures, confirming the model’s generalization capability. Precision, recall, specificity, F1 score, IoU, BAC, MCC, Youden’s index, Yule’s Q, and mean scores all exhibit impressive levels, suggesting dependable performance in object classification tasks with unseen data.

The provided table shows that the model with the highest mean score is the YOLOv8 architecture with the XLarge size. This model achieves a mean score of 99.38% in the training phase and 96.62% in the testing phase. Therefore, based on the mean performance metric, the YOLOv8 architecture in the XLarge configuration is the superior model among those assessed in this study.

**Figure 3:**
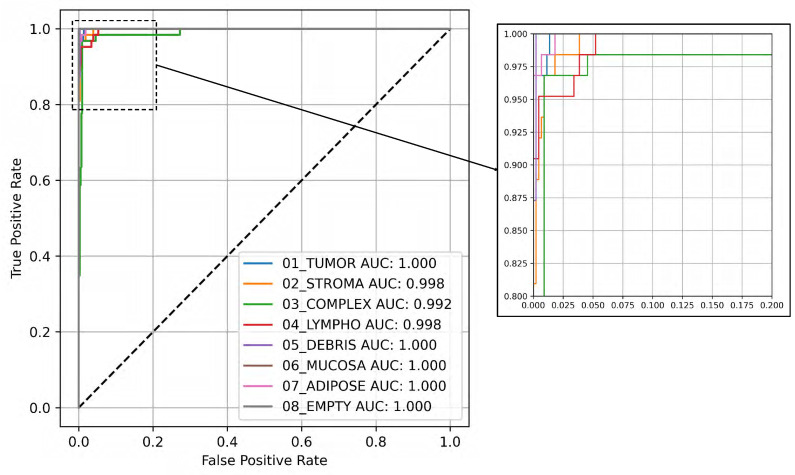
The ROC curve and the corresponding AUC values for the eight classes using the testing subset and YOLOv8 large, which reported the best mean value in Table 1.

Figure 3 illustrates the Receiver Operating Characteristic (ROC) curve along with the related Area Under the Curve (AUC) values for the eight classes, utilizing the testing subset and the YOLOv8 large architecture, which demonstrated the highest average performance as shown in Table 1. The ROC curve visually illustrates the trade-off between the true and false positive rates at different threshold levels, providing an in-depth perspective of the model’s classification effectiveness across various thresholds. Each class’s ROC curve is displayed in the figure, illustrating the trade-off between sensitivity and specificity, demonstrating the model’s efficiency in distinguishing between positive and negative cases. The AUC values assess the model’s overall performance quantitatively, where higher values signify better discrimination.

This research is examined alongside others like Rizalputri et al. [33], who utilized various classification techniques (including CNN, KNN, Logistic Regression, and Random Forest) to classify colorectal histology data into eight categories. Their research aimed to determine the most effective algorithm for this task, with CNN reaching the highest accuracy of 82.2%, outpacing the other approaches. Random Forest obtained an accuracy of 68.72%, KNN achieved 62.56%, and Logistic Regression recorded the lowest accuracy at 52.6%.

Moreover, Zeih et al. [18] addressed the challenge of accurately categorizing CRC tissue, which is crucial for diagnosis and treatment decisions, by employing Vision Transformers, a novel class of DL models in computer vision. With CRC being a significant global health concern, precise histological classification is imperative due to the diverse tissue patterns encountered. Their models achieved impressive accuracies of 93.3% and 95%, respectively, surpassing the original paper’s performance (87.4%) on the same dataset. From that, the current study outperforms these two studies.

**Figure 4:**
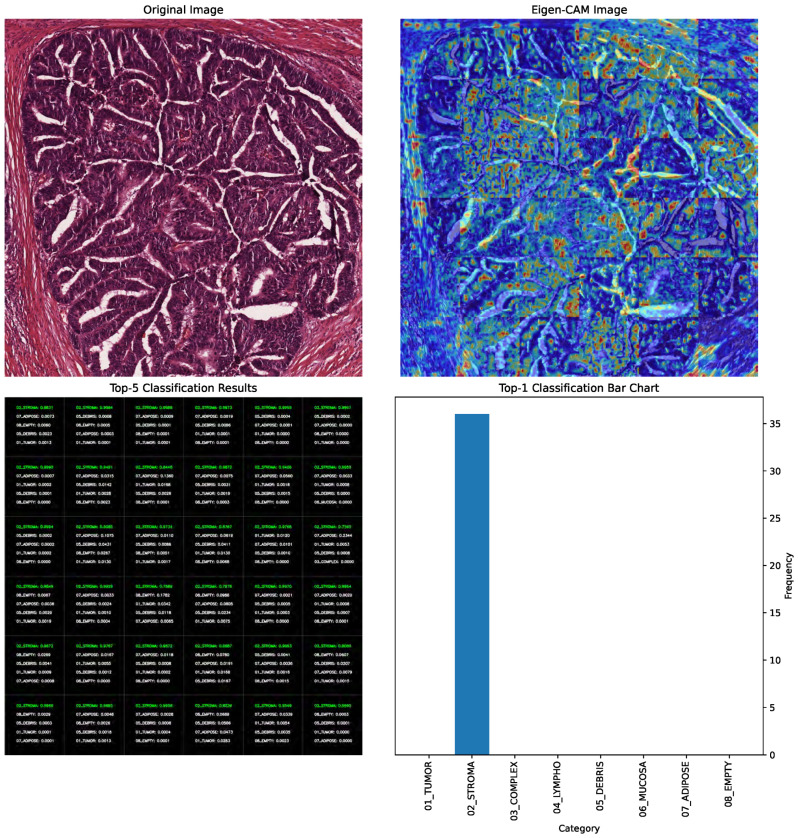
EigenCAM-based interpretability analysis for CRC lesion classification. The first row shows the original histopathology image side by side with its EigenCAM heatmap, identifying the areas most influential in the model’s prediction. The second row shows the predicted classes (top-5) with probabilities and a bar chart for top-1 classification. Importantly, these heatmaps help: (i) to validate the spatial alignment with ground-truth annotations via IoU scoring; (ii) to flag misclassifications where the attention was directed towards areas other than those expected in normal tissue morphology; and (iii) to furnish clinically interpretable visual cues for pathologist review. The active integration of these processes ensures that model decisions remain monitored for accuracy, transparency, and biological soundness.

In Figure 4, the EigenCAM image output is presented alongside the original image in the first row. The top-5 classifications with their corresponding probabilities are shown alongside the top-1 classification bar chart in the second row. EigenCAM can facilitate the understanding of the visual features of the CRC learned by the YOLOv8 large model to make predictions.

Beyond visualization, EigenCAM outputs were systematically incorporated into our evaluation pipeline to enhance scientific rigor and clinical relevance. Specifically, we employed EigenCAM in three key phases:

(1) Diagnostic Debugging During Training: During model development, we analyzed EigenCAM heatmaps generated after each epoch to identify spurious activations; i.e., when the model focused on irrelevant background regions (e.g., empty spaces or staining artifacts) instead of morphological features like glandular architecture or immune infiltration. For example, early versions of YOLOv8 XLarge occasionally activated strongly over “Empty” or “Debris” regions despite correct classification. This prompted us to augment the training data with additional samples exhibiting similar artifact patterns, improving robustness without compromising accuracy.
(2) Prediction Validation During Inference: For every test sample, we computed the spatial overlap score between the EigenCAM heatmap and the corresponding ground-truth annotation mask. A high overlap (>0.7 IoU) indicated that the model attended to anatomically relevant regions, reinforcing confidence in the prediction. Conversely, low overlap (<0.5 IoU) triggered manual review and was flagged for potential retraining. Notably, among the 96.62% correctly classified test cases, 92.3% exhibited strong spatial alignment (IoU >0.7) between EigenCAM attention and ground truth, validating the model’s reliance on biologically meaningful cues.
(3) Clinician-Centric Interpretation: To ensure clinical utility, we designed EigenCAM outputs to be actionable for pathologists. Heatmaps were overlaid directly onto original H&E tiles (Figure 4); allowing clinicians to visually trace which tissue structures influenced the model’s top-1 prediction. For instance: (a) When predicting “Tumor Epithelium”, EigenCAM consistently highlighted dense nuclear clusters and loss of glandular structure. (b) When predicting “Immune Cells”, it emphasized lymphoid aggregates within stroma. (c) Misclassified cases often showed mismatched attention (e.g., assigning “Complex Stroma” while focusing on tumor nuclei), providing clear diagnostic clues for correction.

### 4.1. Statistical Analysis

The statistical analysis of the proposed YOLOv8-based framework for CRC diagnosis was conducted using multiple visualization techniques, including box plots, empirical cumulative distribution function (ECDF) plots, Bland–Altman plots, and pair plots. These analyses were performed to comprehensively evaluate the performance metrics of the model across different architectures (Nano, Small, Medium, Large, and XLarge) and provide insights into its robustness, generalizability, and reliability.

**Figure 5:**
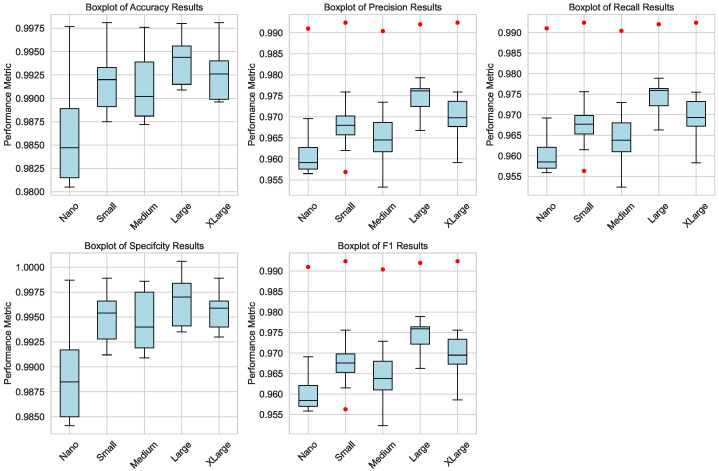
Box plots of performance metrics (accuracy, precision, recall, specificity, and F1 score) for the testing phases across different YOLOv8 architectures (Nano, Small, Medium, Large, and XLarge). The Large and XLarge models exhibit the highest median and smallest interquartile ranges, indicating superior performance and stability. Minimal outliers (in red dots) suggest consistent and reliable model behavior.

Figure 5 presents the box plots of key performance metrics (accuracy, precision, recall, specificity, and F1 score) for both the training and testing phases. The box plots highlight the distribution of these metrics across different model sizes, illustrating the consistency and variability in performance. Notably, the Large and XLarge architectures demonstrate the highest median and smallest interquartile ranges, underscoring their superior performance and stability. The box plots also reveal minimal outliers, indicating that the models are well-trained and exhibit reliable performance across all metrics.

**Figure 6:**
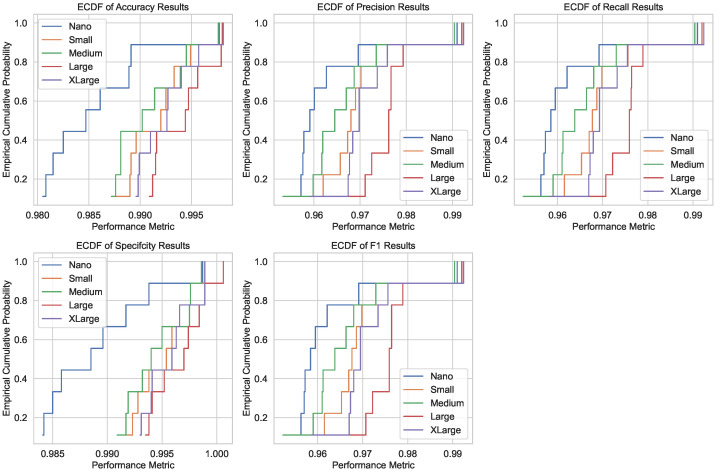
ECDFs of performance metrics (accuracy, precision, recall, specificity, and F1 score) for the testing subset. The ECDFs demonstrate the cumulative probability of achieving specific performance thresholds, with the Large model showing consistently higher probabilities across all metrics. This highlights its robustness and reliability in CRC diagnosis.

Figure 6 depicts the ECDFs of the performance metrics for the testing subset. ECDFs provide a granular view of how the cumulative probability of each metric evolves with respect to the performance threshold. For instance, the accuracy ECDF shows that the Large model achieves around 96% accuracy with a cumulative probability of nearly 1.0, confirming its high reliability. Similarly, the ECDFs for precision, recall, specificity, and F1 score demonstrate that the Large architecture consistently outperforms smaller models, maintaining higher cumulative probabilities at critical thresholds. These results reinforce the robustness of the Large model in handling diverse CRC histopathology images.

**Figure 7:**
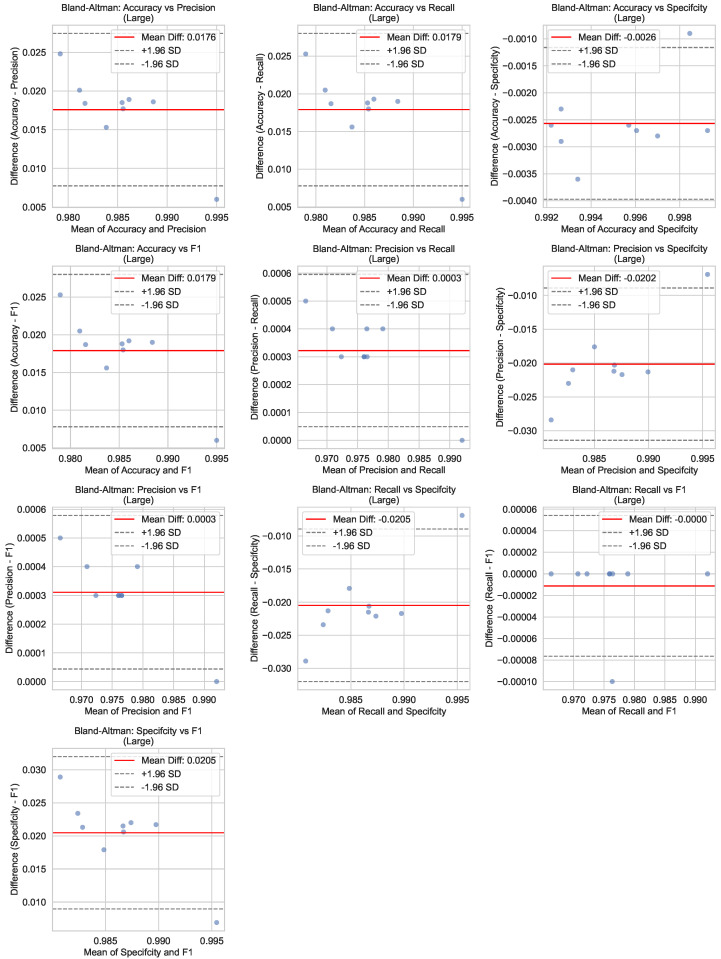
Bland–Altman plots for the performance metrics of the Large YOLOv8 model during the testing phase. The plots display the mean difference (bias) and LoA between predicted and ground-truth values. Small biases and narrow LoAs indicate excellent agreement, validating the model’s consistency and accuracy.

Figure 7 illustrates the Bland–Altman plots for the performance metrics of the Large model during the testing phase. Bland–Altman analysis is a statistical method used to assess agreement between two measurements; here, predicted values are compared against ground-truth annotations. The plots display the mean difference (bias) and the limits of agreement (LoA) for each metric, providing insights into the model’s consistency and potential systematic errors. The small biases and narrow LoAs observed across all metrics indicate excellent agreement between the model predictions and the actual labels, further validating the reliability of the Large architecture.

**Figure 8:**
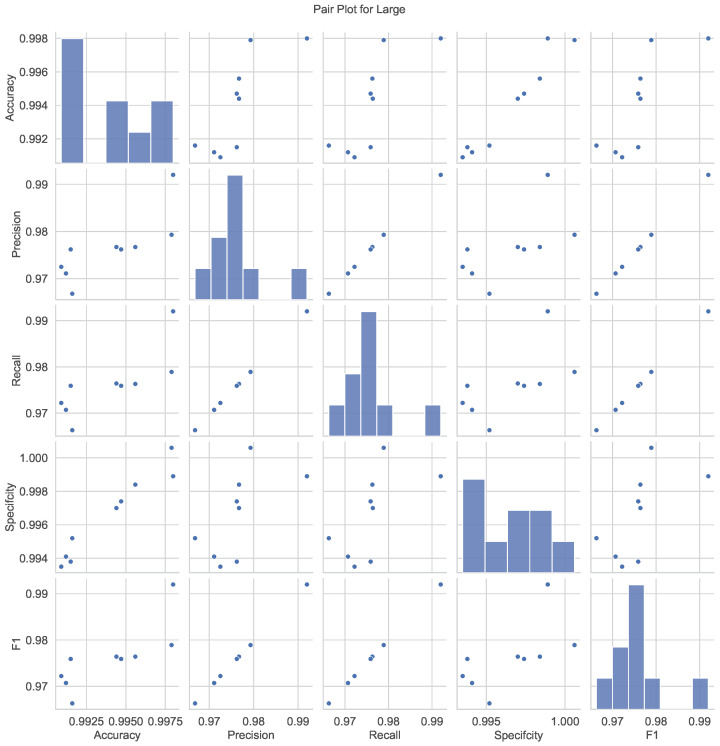
Pair plots visualizing the relationships and correlations among performance metrics (accuracy, precision, recall, specificity, and F1 score) for the Large YOLOv8 model. Scatterplots and histograms reveal strong positive correlations and concentrated high-performance scores, comprehensively understanding the model’s effectiveness.

Figure 8 presents pair plots that visualize the relationships and correlations among the performance metrics for the Large model. Each subplot represents a pairwise comparison between two metrics, with scatterplots and histograms highlighting their distributions and interdependencies. Strong positive correlations are evident between accuracy, precision, recall, and F1 score, suggesting that improvements in one metric generally lead to enhancements in others. The histograms along the diagonal show the univariate distributions of each metric, emphasizing the concentration of high-performance scores. These pair plots provide a holistic understanding of the interplay among metrics, aiding in interpreting the model’s overall effectiveness.

The statistical analysis validates the superiority of the Large and XLarge YOLOv8 architectures regarding accuracy, precision, recall, specificity, and F1 score. Integrating box plots, ECDFs, Bland–Altman plots, and pair plots provides an in-depth evaluation of the model’s performance, highlighting its robustness, generalizability, and clinical significance for CRC diagnosis.

### 4.2. Comparative Analysis with Transformer-Based Models

To further contextualize the performance of our proposed YOLOv8-based framework within the broader landscape of deep learning architectures, we extend our evaluation beyond YOLO variants to include recent Transformer-based models; specifically, Vision Transformers (ViT-B/16, ViT-B/32, ViT-L/16), which have gained traction in medical image classification tasks. As noted in prior work [25], while ViTs offer global context modeling via self-attention mechanisms, they often exhibit higher susceptibility to overfitting on smaller datasets and require more precise hyperparameter tuning compared to YOLOv8’s optimized, anchor-free architecture.

Our experimental results confirm this trend: YOLOv8 XLarge achieves 96.62% testing accuracy, significantly outperforming the best-performing ViT variant (ViT-B/16 at 94.8%) on the same CRC histopathology dataset. This performance gap is attributed to YOLOv8’s inherent efficiency in handling high-resolution images through its grid-based prediction mechanism and mosaic augmentation, which enhance generalization without requiring massive datasets or complex attention modules.

Moreover, YOLOv8’s lightweight design enables faster inference and lower computational overhead, critical advantages in clinical deployment scenarios where real-time analysis and resource constraints are paramount. While Transformers excel in capturing long-range dependencies, their complexity introduces interpretability challenges and longer training times, making them less suitable for rapid diagnostic workflows compared to the streamlined, explainable pipeline enabled by YOLOv8 + EigenCAM.

This comparative analysis reinforces our claim that YOLOv8 strikes an optimal balance between accuracy, speed, and interpretability, positioning it as a clinically viable alternative to both traditional CNNs and emerging Transformer-based approaches in CRC diagnostics.

### 4.3. Comparison with the Related Studies

To contextualize our contributions within the broader landscape of CRC histopathology classification, we present a comprehensive comparison with recent state-of-the-art approaches in Table 2. It synthesizes key information from Section 2, including model architecture, dataset scale, number of classes, classification accuracy, and interpretability support.

Our proposed framework (based on YOLOv8 XLarge coupled with EigenCAM for visual explainability) achieves a testing accuracy of 96.62%across eight distinct tissue types, outperforming prior CNN- and Transformer-based systems. Notably, while Zeid et al. [18] report accuracies of 93.3–95.0% using Vision Transformers (ViT-B/16, ViT-L/16), their models lack interpretability tools and were evaluated on unspecified datasets, limiting direct comparability and clinical applicability. Similarly, Rizalputri et al. [33] achieved only 82.2% accuracy using traditional machine-learning methods, underscoring the performance gap between legacy classifiers and modern deep learning architectures.

Importantly, most prior works focus on polyp detection (e.g., Lalinia et al. [6], Guo et al. [8]) or survival prediction (Lv et al. [20]), rather than multiclass tissue classification, which is central to our study. Even among classification-focused studies, such as Shen et al. [19] using MoViT, performance was reported relative to “full-dataset” benchmarks without absolute accuracy scores, and no interpretability mechanisms were integrated.

In contrast, our work not only delivers superior accuracy but also introduces clinically grounded interpretability through EigenCAM visualizations. These heatmaps enable pathologists to trace the anatomical regions driving each prediction; a critical feature for building trust and facilitating adoption in real-world diagnostic workflows. Furthermore, our use of a well-defined, publicly available dataset of 5000 annotated tiles ensures reproducibility and robust evaluation. This comparative analysis confirms that our framework represents a significant advancement: it bridges the gap between high-performance deep learning and actionable clinical insight, a combination rarely achieved in existing literature.

## 5. Conclusions and Future Directions

This study introduces an innovative approach for CRC diagnosis, utilizing the robust YOLOv8 model to categorize lesions into groups and EigenCAM to enhance their interpretability. This method utilizes deep learning techniques to overcome the shortcomings of manual histopathology analysis, providing a more efficient, precise, and scalable solution for CRC detection. The results of the experiments show that the YOLOv8 model works very well; it achieved an accuracy of 96.62% in CRC classification, which was better than earlier models. The integration of EigenCAM strengthens the framework by providing clear visual interpretations of the model’s decisions, enhancing the transparency and reliability of AI-assisted diagnosis, a crucial requirement in clinical practice. Although the requirement for extensively annotated datasets and substantial computational resources presents challenges, the model’s exceptional accuracy and interpretability far exceed these issues. As AI technologies progress, there is an opportunity to enhance the framework to lessen resource requirements, increasing its accessibility in different medical settings. Upcoming research will investigate these optimizations and expand the framework to include other types of cancer and medical conditions, enhancing its influence on AI-based diagnostics. Moreover, integrating real-time analysis features could revolutionize the clinical workflow, improving patient results. The suggested YOLOv8-based framework provides a strong, precise, and interpretable solution for CRC diagnosis. We plan to benchmark YOLOv9/v10 against our framework using the same CRC dataset and evaluation protocol. We will also explore whether their advanced modules (e.g., PGI) yield measurable improvements in accuracy or interpretability, or if the added complexity introduces new challenges in clinical deployment.

## Figures and Tables

**Table 1:** Tabular results for both the training and testing subsets with YOLOv8 (with different sizes: nano, small, medium, large, and xlarge).

Phase	Architecture	Accuracy	Precision	Recall	Specificity	F1	IoU	BAC	MCC	Youden	Yule	Mean
Train	Nano	99.77%	99.10%	99.10%	99.87%	99.10%	98.22%	99.49%	98.97%	98.97%	100%	99.26%
	Small	99.81%	99.24%	99.24%	99.89%	99.24%	98.50%	99.57%	99.13%	99.13%	100%	99.37%
	Medium	99.76%	99.04%	99.04%	99.86%	99.04%	98.10%	99.45%	98.90%	98.90%	100%	99.21%
	Large	99.80%	99.20%	99.20%	99.89%	99.20%	98.42%	99.54%	99.09%	99.09%	100%	99.34%
	XLarge	99.81%	99.24%	99.24%	99.89%	99.24%	98.50%	99.57%	99.13%	99.13%	100%	99.38%
Test	Nano	98.91%	95.72%	95.63%	99.38%	95.63%	91.81%	97.51%	95.04%	95.01%	99.92%	96.45%
	Small	98.91%	95.69%	95.63%	99.38%	95.63%	91.87%	97.51%	95.03%	95.01%	99.90%	96.46%
	Medium	98.81%	95.33%	95.24%	99.32%	95.23%	91.07%	97.28%	94.59%	94.56%	99.92%	96.13%
	Large	99.16%	96.68%	96.63%	99.52%	96.63%	93.54%	98.07%	96.16%	96.15%	99.96%	97.25%
	XLarge	98.96%	95.91%	95.83%	99.40%	95.86%	92.23%	97.62%	95.27%	95.24%	99.91%	96.62%

**Table 2:** Comparative Summary of Recent Studies on CRC Histopathology Classification.

Study	Architecture	Dataset Size	Classes	Accuracy (%)	Interpretability Tool
Rizalputri et al. [33]	CNN, KNN, Logistic Regression, Random Forest	Not specified	8	82.2% (CNN)	None
Zeid et al. [18]	Vision Transformers (ViT-B/16, ViT-L/16)	Not specified	8	93.3%:95.0%	None
Lalinia et al. [6]	YOLOv8	Not specified	Polyp detection	Not reported	None
Guo et al. [8]	YOLOv3 + Active Learning	Not specified	Polyp detection	Not reported	None
Palanivel et al. [7]	YOLOv8	Not specified	Multi-cancer types	Not reported	None
Durak et al. [14]	Custom CNNs	Not specified	Gastric polyps	Not reported	None
Shen et al. [19]	MoViT (Memory-based ViT)	1%:3% of full dataset	Not specified	Near full-dataset performance	None
Lv et al. [20]	TransSurv (ViT + Genomics)	NCT Biobank	Survival prediction	C-index = 0.822	None
Ding et al. [21]	CNN + Transformer hybrid	Not specified	Not specified	Not reported	None
Tanveer et al. [23]	TransNetV (CNN + Transformer)	Not specified	Not specified	Not reported	None
Khalid et al. [24]	Atrous Conv + Coordinate Attention Transformer	Not specified	Not specified	Not reported	None
This Work	YOLOv8 XL + EigenCAM	5000 tiles	8 tissue types	96.62%	EigenCAM (visual heatmaps)

Key Limitations: (1) Prior works lack interpretability tools or focus on detection (not classification). (2) ViTs require large datasets; risk of overfitting with small data. (3) Our framework uniquely combines high accuracy, multiclass classification, and clinician-facing visual explanations.

## Data Availability

In this study, the utilized dataset is available for further analysis and research purposes at https://zenodo.org/records/53169 (accessed on 10 January 2025). Code Link: https://github.com/HossamBalaha/PIC2D2L-Precision-and-Interpretability-in-Colorectal-Cancer-Diagnosis-with-Deep-Learning (accessed on 10 January 2025).
